# Axl promotes intracranial aneurysm rupture by regulating macrophage polarization toward M1 *via* STAT1/HIF-1α

**DOI:** 10.3389/fimmu.2023.1158758

**Published:** 2023-05-08

**Authors:** Yongquan Han, Gaozhi Li, Zeyu Zhang, Xiaohua Zhang, Bing Zhao, Hua Yang

**Affiliations:** ^1^ Department of Neurosurgery, the Affiliated Hospital of Guizhou Medical University, Guiyang, China; ^2^ Department of Neurosurgery, Renji Hospital, Shanghai Jiaotong University School of Medicine, Shanghai, China

**Keywords:** intracranial aneurysm, rupture, Axl, macrophage polarization, STAT1, HIF-1α

## Abstract

**Background:**

Macrophage infiltration and polarization are crucial for the pathogenesis of intracranial aneurysm (IA) rupture. Axl, a receptor tyrosine kinase, is involved in inflammation and efferocytosis in multiple organs. Upregulated soluble Axl in cerebrospinal fluid (CSF) and plasma is correlated with intracranial aneurysm rupture. This study aimed to investigate the role of Axl in IA rupture and macrophage polarization.

**Methods:**

Male C57BL/6J mice were used to induce IA. The level of Axl from control vessels and unruptured and ruptured IA samples was detected. In addition, the relationship between Axl and macrophages was confirmed. The pathway of Axl-mediated macrophage polarization was explored after IA induction *in vivo* and in bone marrow-derived macrophages (BMDMs) stimulated by LPS/IFN-γ *in vitro*. The animals were randomized into three groups and treated intraperitoneally with the vehicle, selective AXL antagonist R428, and recombinant mouse growth arrest-specific 6 (rmGas6) for 21 consecutive days. Then, we evaluated the influence of Axl on IA rupture by administrating R428 to inhibit or rmGas6 to activate the Axl receptor *in vivo*.

**Results:**

Compared with that in normal vessels, Axl expression was significantly upregulated in unruptured IA samples. The ruptured IA tissue exhibited significantly higher expression of Axl than the unruptured IA tissue. Axl and F4/80 were coexpressed in IA tissue and LPS/IFN-γ-stimulated BMDMs. R428 treatment significantly reduced the rate of M1-like macrophage infiltration and IA rupture. In contrast, rmGas6 treatment promoted M1 macrophage infiltration and IA rupture. Mechanistically, R428 inhibited the phosphorylation of Axl and STAT1 and the expression of hypoxia-inducible factor-1α (HIF-1α) and decreased the levels of IL-1β, NOS2, and MMP9 in LPS/IFN-γ-stimulated BMDMs. rmGas6 promoted the phosphorylation of Axl and STAT1 and the expression of HIF-1α. In addition, STAT1 knockdown abolished Axl-mediated M1 macrophage polarization.

**Conclusion:**

The inhibition of Axl reduced macrophage polarization toward the M1 phenotype *via* the STAT1/HIF-1α signaling pathway and prevented IA rupture in mice. This finding suggests that pharmacological inhibition of Axl might be used to prevent the progression and rupture of IA.

## Introduction

The prevalence of unruptured intracranial aneurysm (IA) is estimated to be approximately 3.2% in the population around the world (1). Unruptured IAs are more frequently detected with the modern imaging modalities used in clinical practice. Ruptured IAs have a high rate of mortality and morbidity and cause a lifelong cognitive deficit for those who survive (2, 3). Most IAs are found incidentally and need preventive treatment to prevent rupture. Currently, there are two main treatment modalities, clipping and coiling, which are associated with high procedure-related complications (4). Therefore, pharmacological therapy may be a potential way to prevent IA rupture with minimal risk (5, 6).

IA is associated with complicated pathological changes characterized by an inflammatory response triggered by abnormal hemodynamic stresses in cerebral arteries (7–9). Recent studies have shown that the infiltration of the proinflammatory macrophage subtype, classified as M1-like macrophages, can exacerbate pathogenesis and promote IA rupture (10, 11). Macrophage-mediated cellular and molecular inflammation are crucial in aneurysm rupture.

A family of receptor tyrosine kinases, Tyro3, Axl, and Mer (TAM), expressed on macrophages, mediates the process of efferocytosis, phagocytosis, and the inflammatory response (12–15). In contrast to Tyro3 and Mer, the expression of Axl is stimulated by proinflammatory mediators in macrophages and dendritic cells (16, 17). In addition, the extracellular domain of Axl is proteolytically cleaved and released, which is called soluble Axl (solAxl). The upregulated solAxl in both cerebrospinal fluid (CSF) and plasma is correlated with IA rupture (18). However, whether Axl regulates the macrophage response in aneurysm biopsies in a manner that causes IA rupture needs to be investigated. Moreover, the detailed mechanism of Axl in the process and pathogenesis of IA is still unclear.

To clarify the role of Axl in IA rupture, we evaluated Axl expression and its relationship with macrophage infiltration in mouse IA models. We further determined whether Axl promotes macrophage polarization toward the M1 phenotype by activating STAT1/HIF-1α signaling, which promotes aneurysm rupture. This study potentially provides a new therapeutic target and may facilitate the design of pharmacological treatments for IAs.

## Materials and methods

### The induction of the mouse IA model

Animal experimental protocols were approved by the Institutional Animal Care and Use Committee of Shanghai Jiaotong University. Male C56BL/6J mice (6-8 weeks) were purchased from Charles River Laboratories (Shanghai, China). IA was induced by combining systemic hypertension and elastase injection as previously described (19). Briefly, after being anesthetized by a mixture of 3% isoflurane and O_2_ (1 L/min) on a heating pad, individual mice were subjected to ligation of the left common carotid artery and bilateral posterior branches of the renal artery with 6-0 nylon suture. One week later, the mice were anesthetized as described above and fixed in a stereotaxic frame with a mouse adaptor (RWD, China). According to the coordinates from Mouse Brain Atlas, mice were injected with 35 mU (milli-unit) elastase (E1250, Sigma-Aldrich, USA) through a Hamilton syringe with a 34 G needle (Hamilton, Switzerland) at a rate of 0.2 µl/min into their suitable basal cistern (2.5 mm posterior to the bregma, 1.0 mm right to the middle and 5.3 mm deep to the skull surface). Subsequently, an osmotic pump (A1004, Alzet Osmotic Pump, USA) containing angiotensin II (A1042, APExBIO, USA) was implanted into a subcutaneous pocket between the scapulae to deliver angiotensin II at a rate of 1000 ng/kg/min for systemic hypertension induction. Upon recovery, the mice were fed food containing 8% NaCl and 0.12% BAPN (N-methyl-β-alanineitrile, M27603, Sigma-Aldrich, USA).

### Animal treatment

To investigate the effect of Axl on IA rupture, we administered the selective AXL antagonist R428 and recombinant mouse growth arrest-specific 6 (rmGas6) *in vivo* to inhibit or activate the biological effect of Axl, respectively. Mice that died within 7 days after IA induction were excluded. One week later, the animals were randomized into three groups (n=15 each group) and treated intraperitoneally with vehicle (phosphate-buffered saline, PBS), 75 mg/kg R428 (HY-15150, MCE, USA) or 4 µg/kg rmGas6 (986-GS, R&D Systems, USA) every day for 21 consecutive days. The concentration of R428 administered intraperitoneally was 2 mmol/L. The doses of R428 and rmGas6 were applied according to previous studies (20, 21). Systolic arterial blood pressure (SBP) was measured and recorded from IA induction with tail-cuff measurement (Softron, BP-2010, Japan). All animals were euthanized for tissue harvest 21 days after IA induction.

### Tissue harvest

The mice were monitored daily. Intracranial aneurysm formation was defined as localized outward bulging of the artery wall. After euthanization, the mice were perfused with precooled PBS, 4% paraformaldehyde, and Microfil (MV-120, Flow Tech, USA) sequentially to visualize vessels under a microscope by two researchers in a blinded manner. Intracranial aneurysms were classified as follows: Grade 0, normal artery; Grade 1, artery dilation or tortuosity; Grade 2, unruptured aneurysm; and Grade 3, ruptured aneurysm. Unruptured aneurysms were defined as 1.5 times larger in diameter than the parent artery. An aneurysm with fresh blood clots along the cerebral artery, revealing subarachnoid hemorrhage, was defined as aneurysm rupture.

### Preparation of bone marrow-derived macrophages

Bilateral femurs and tibias were dissected from male C57BL/6J mice and flushed with sterilized PBS. After lysis of red blood cells with Red Blood Cell Lysis Buffer (11814389001, Roche, Switzerland), the remaining nuclear cells were filtered through a 70 µm cell strainer (352350, Corning, USA). BMDMs were diluted to 5×10^5^ cells per milliliter and cultured in Dulbecco’s modified Eagle’s medium (DMEM) containing 10% FBS and 1% penicillin/streptomycin in the presence of 25 ng/ml recombinant murine M-CSF (315-02, Peprotech, USA) for 7 days to induce macrophage maturation at 37°C in a 5% CO_2_ incubator.

### Cell transfection

Cells extracted from bone marrow were small round cells and were plated in a culture dish at a concentration of 5x10^5 cells/ml and incubated with M-CSF stimulation for 7 days. During stimulation with M-CSF, BMDMs became larger with different-shaped antennae. The cell volume reached 70% of the dish area after 7 days of stimulation with M-CSF. Cells were transfected with 100 nM control scramble (6568, CST, USA) or 100 nM STAT1 siRNA (sc-44124, Santa Cruz, USA) using Lipofectamine™ 3000 (L3000001, Invitrogen, USA) according to the manufacturers’ instructions. The transfection efficiency was evaluated by real-time quantitative PCR (RT-qPCR) and Western blotting.

### Induction of M1 macrophage polarization

Mouse BMDMs were cultured in serum-free medium for 6 hours and treated with 100 ng/ml LPS and 20 ng/ml IFN-γ (HY-D1056/HY-P7071, MCE, USA) for 6 hours in complete medium containing 1 µg/ml R428 and 200 ng/ml rmGas6 to inhibit or enhance the phosphorylation of Axl.

Human acute monocytic leukemia THP-1 cells were purchased from Procell (Wuhan, China). THP-1 cells were cultured in RPMI-1640 medium containing 10% (v:v) fetal bovine serum and 1% (v:v) penicillin-streptomycin solution. THP-1 cells were differentiated into macrophages by incubation with 0.5 μM 12-O-tetradecanoylphorbol 13-acetate/PMA (A606759, Sangon Biotech, China) for 12 hours. After washing with PBS, the THP-1 macrophages were primed with 100 ng/ml LPS and 20 ng/ml IFN-γ to polarize toward M1 macrophages and treated with or without R428 and Gas6 (HY-P77668, MCE, US) for 6 hours.

### Hematoxylin and eosin and Masson’s trichrome staining

Individual tissue samples were fixed in 4% paraformaldehyde overnight. Then, the tissue samples were processed, paraffin-embedded, and sectioned manually, as described previously. The tissue sections (10 µm) were stained with HE (G1120, Solarbio, China) and Masson (G1340, Solarbio, China) according to the manufacturers’ instructions.

### Immunofluorescence

The fixed vascular tissues were dissected and frozen in OCT compound. The crystal tissue sections (10 μm) were cultured with 0.3% Tween-100 for 30 min and blocked with 5% donkey serum for 1 h at room temperature. Similarly, the coating coverslips (WHB-12-CS, WHB Scientific, China) on which cells were plated were fixed and treated as described above. These samples were incubated overnight at 4°C with primary antibodies against Axl (13196, Proteintech), CD86 (14-0862-82, Invitrogen, USA), pSTAT1 (9167, Abcam) and F4/80 (71299, CST). After being washed with PBS, the slides were exposed to the indicated fluorophore-labeled secondary antibodies (ab150075, ab150165, Abcam, USA) at room temperature for 2 hours and nuclear-stained 4′,6-diamidino-2-phenylindole (DAPI) (C1002, Beyotime, China). The fluorescent signals were examined and photo imaged under a laser scanning confocal microscope (LSM800, Zeiss, Germany).

### Quantitative real-time -PCR

Total RNA was extracted from individual cell samples and purified using an RNA extraction kit (AG21017, Accurate Biology, China) according to the manufacturers’ instructions. After RNA concentration quantitation through a spectrophotometer (ND-1000, Nanodrop Technologies, USA), RNA samples were individually reverse-transcribed into cDNA using PrimeScript™ RT Master Mix (RR036A, Takara, Japan). The relative levels of targeted gene mRNA transcripts to the control GAPDH were quantified by TB Green^®^ Premix Ex Taq™ II (RR820, Takara, Japan) and specific primers in a Real-Time PCR System (SterpOne Plus, Thermo, USA). The PCRs were performed in duplicate, and the data were analyzed by the 2^-ΔΔCt^ method. The primer sequences are listed below.

*Axl* (Forward: TGA GCC AAC CGT GGA AAG AG; Reverse: AGG CCA CCT TAT GCC GAT CTA)

*Il-1β* (Forward: CAC TAC AGG CTC CGA GAT GAA CAA C; Reverse: TGT CGT TGC TTG GTT CTC CTT GTA C)

*Nos2* (Forward: AGC GAG GAG CAG GTG GAA; Reverse: GGA AAA GAC TGC ACC GAA GAT ATC)

*Mmp9* (Forward: GTA CTC GAC CTG TAC CAG CG; Reverse: TCA GGG CGA GGA CCA TAG AG)

*Gapdh* (Forward: GGG GAG CGA GAT CCC TCC AAA ATC AAG TGG GG; Reverse: GGG TCA TGA GTC CTT CCA CGA TAC CAA AGT TG).

### Western blot

The different groups of cells were lysed with radioimmunoprecipitation assay (RIPA) buffer (P0013, Beyotime, China) with a dose of 100 µl RIPA per 1×10^6^ cells. After being boiled with sample buffer at 99°C for 5-10 mins, the cell lysates were separated by SDS-PAGE and transferred to PVDF membranes. After being blocked with 5% nonfat milk dissolved in TBST, the membranes were incubated at 4°C overnight with primary antibodies against Axl (bs-5180R, Bioss, China), pAxl (bs-5181R, Bioss, China), STAT1 (14994S, CST, USA), pSTAT1 (7649S, CST, USA), HIF-1α (ab179483, Abcam, USA), IL-1β (CY5087, Abways, China), NOS2 (CY5993, Abways, China), MMP9 (CY5205, Abways, China) and β-actin (81115, Proteintech, China). The membranes were exposed to the indicated HRP-conjugated secondary antibodies at room temperature for 1 hour and visualized with ECL through an imaging system (Tanon, China).

### Statistical analysis

Continuous variables were reported as the mean ± standard deviation and analyzed using GraphPad Software (v9.0.1). The continuous variants among multiple groups were compared by one-way ANOVA and *post hoc* test. The rates of aneurysm rupture in the different groups of mice are presented as frequencies or percentages and were analyzed with Fisher’s exact test. The pathological grade data were converted into rank data and tested with the Kruskal-Wallis method. The systolic blood pressure data were analyzed with two-way ANOVA. The survival analysis was performed with the log-rank test. A P value of < 0.05 was considered statistically significant.

## Results

### IA was induced in mice

**Figure 1:**
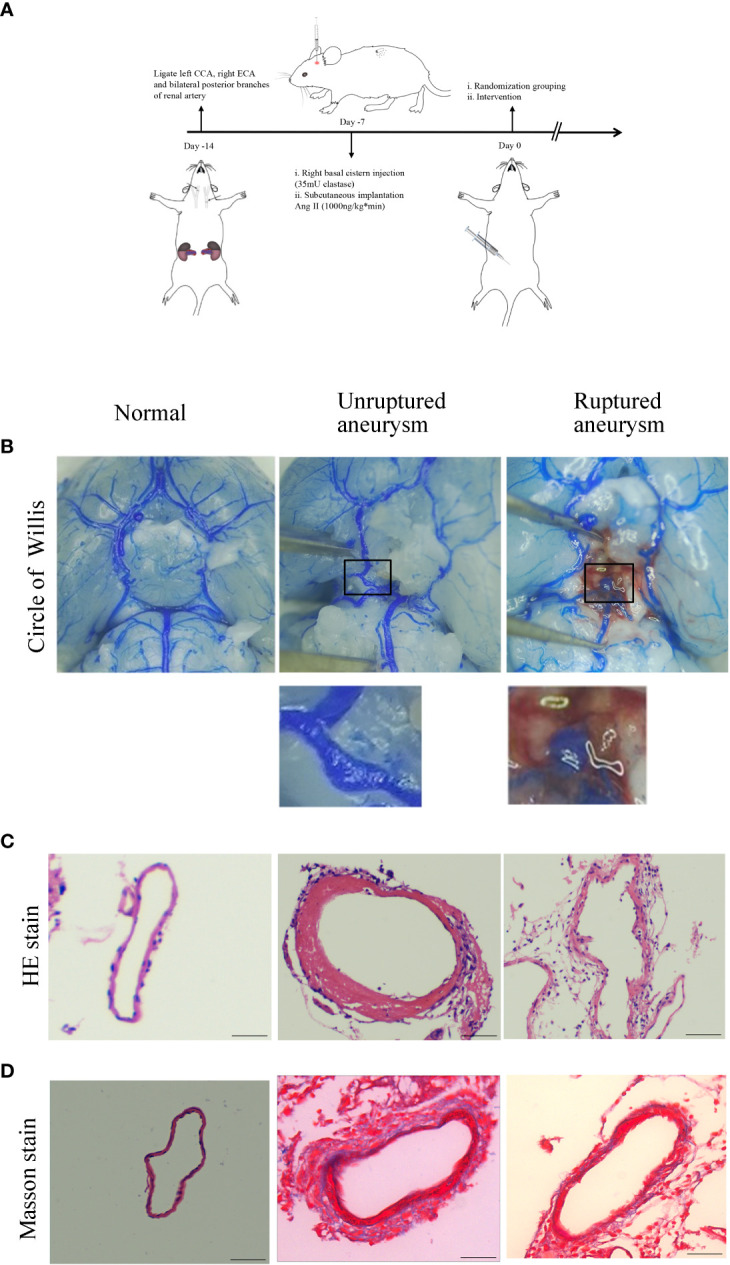
Intracranial aneurysm was established in mice. **(A)** Diagram of IA induction. **(B)** Representative images of normal artery, unruptured aneurysm, and ruptured aneurysm with subarachnoid hemorrhage. **(C)** Hematoxylin and eosin (H&E) staining showed scattered arranged cells in the mouse model of aneurysm. **(D)** Masson’s trichrome staining showed the destruction of vessel walls in the aneurysm. Scale bars=50 μm.

To explore the effect of Axl on the pathogenic process of IA, we established a mouse model of IA (**Figure 1A**). Of the 60 mice, 15 died within 7 days after IA induction for unknown reasons after surgery and were excluded. Representative images of normal arteries, unruptured aneurysms, and ruptured aneurysms in the circle of Willis are shown in **Figure 1B**. To ascertain the pathological changes in IA, hematoxylin-eosin staining (HE) and Masson’s trichrome staining were performed. Compared to normal vessels, HE staining showed thickening of the vascular media and disordered and scattered smooth muscle cells in unruptured aneurysms and thinning of the vessel wall in ruptured aneurysms (**Figure 1C**). Masson’s trichrome staining revealed the disconnection of elastic fibers in the vessel of the aneurysm (**Figure 1D**).

### Upregulated Axl expression was associated with M1 macrophage infiltration in aneurysm lesions

**Figure 2:**
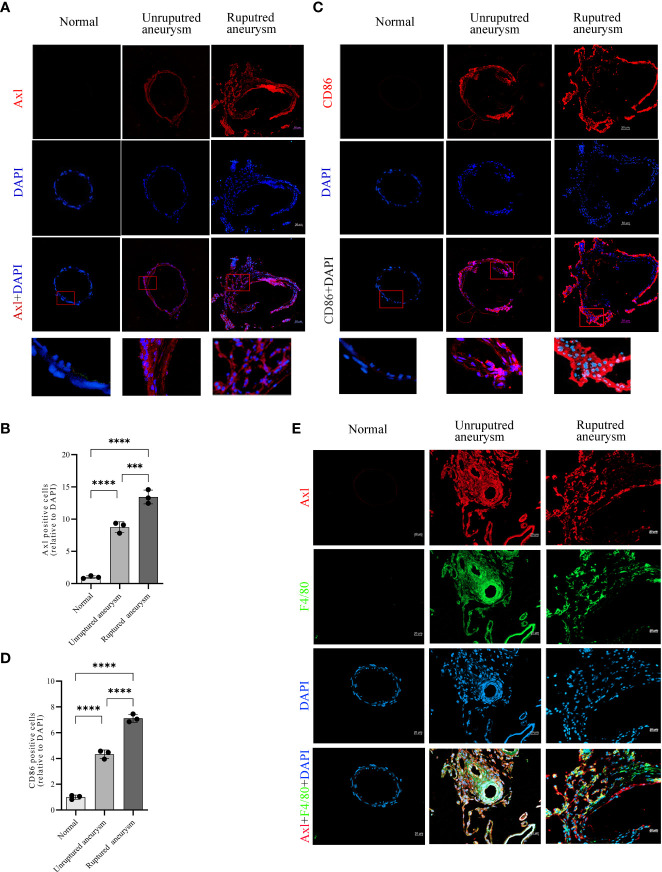
Increased expression of Axl and M1 macrophage infiltration in unruptured and ruptured aneurysm sections compared to normal cerebral artery. Representative immunofluorescence images of Axl **(A)** and CD86 **(C)** expression in normal arteries and unruptured and ruptured aneurysms. Quantitative analysis of Axl **(B)**- and CD86 **(D)**-positive cells. Scale bar = 20 μm **(E)**, Axl (red) and F4/80 (green) coexpressed in aneurysm walls. ***P<0.001, ****P<0.0001.

Immunofluorescence revealed upregulated Axl expression in unruptured aneurysms compared with normal arteries (34.5% vs. 3.9%, P<0.0001) and ruptured aneurysms (53.0% vs. 34.5%, P<0.001; **Figures 2A, B**). Simultaneously, the results also revealed an increase in CD86^+^ (a marker of M1 macrophages) cells in unruptured aneurysms compared with normal arteries (48.0% vs. 11.1%, P<0.0001) and a further increase in ruptured aneurysms (78.8% vs. 48.0%, P<0.0001; **Figures 2C, D**). Double immunofluorescence of IA tissue sections showed that Axl and F4/80 were colocalized in IA tissues (**Figure 2E**). Costaining of Axl and SM22α is shown in **Supplementary Figure S1**.

### Axl activation promoted M1-like macrophage polarization *in vitro*

**Figure 3:**
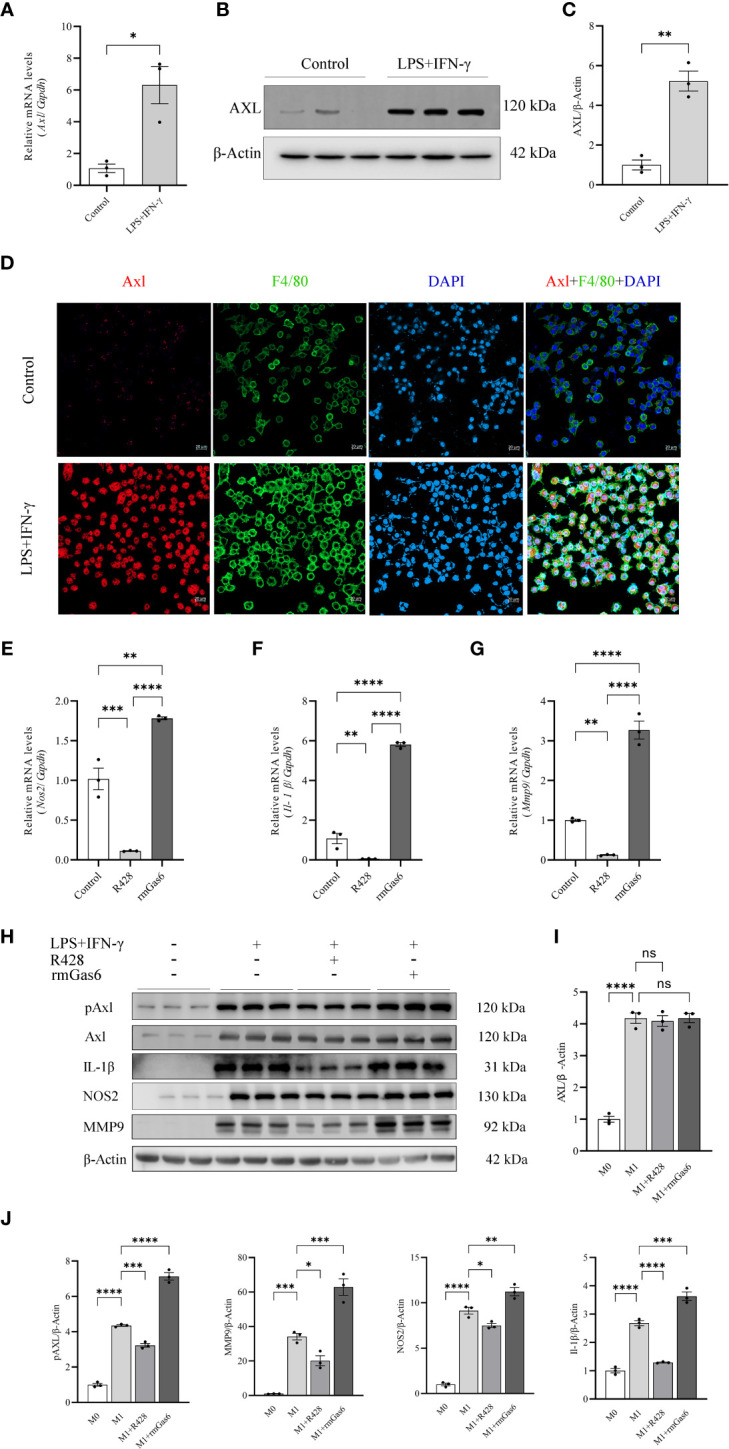
Upregulated Axl promoted M1 macrophage polarization in BMDMs. LPS+IFN-γ increased Axl expression in BMDMs, and inhibition of Axl phosphorylation decreased the levels of inflammatory mediators in LPS+IFN-γ-induced BMDMs. LPS+IFN-γ stimulated Axl transcripts **(A)** and protein in BMDMs **(B, C)**. Representative immunofluorescent images of Axl (red) expression in control and LPS+IFN-γ-stimulated BMDMs labeled with F4/80 (green) **(D)**. qRT-PCR analysis of *IL-1β*, *Nos2*, and *MMP9* expression in LPS+IFN-γ-induced BMDMs treated as indicated **(E–G)**. Western blot analysis of IL-1β, NOS2, and MMP-9 in LPS+IFN-γ-induced BMDMs treated as indicated **(H–J)**. ns, not significant, ^*^P<0.05, ^**^P< 0.01, ^***^P< 0.001, ^****^P< 0.0001.

To further explore the relationship between Axl expression and M1-like macrophage polarization, BMDMs and THP-1 cells were stimulated with or without LPS/IFN-γ (100/20 ng/ml) for 6 hours. LPS/IFN-γ stimulation induced *Axl* expression at the mRNA (P<0.05, **Figure 3A**) and protein levels (**Figures 3B, C**). Axl colocalized with F4/80 in LPS/IFN-γ-stimulated BMDMs (**Figure 3D**). LPS/IFN-γ-primed THP-1 macrophages exhibited increased Gas6 (P<0.01) and Axl (P<0.01) expression and pAxl expression (P<0.0001) (**Supplementary Figure S2**). BMDMs were treated with vehicle, R428 and rmGas6. Compared with the control treatment, R428 treatment inhibited *Il-1β* (P<0.01), *Nos2* (P<0.001), and *Mmp9* (P<0.01) mRNA expression in M1-like macrophages (**Figures 3E–G**). Conversely, rmGas6 treatment had the opposite effect on gene transcription in BMDMs (*Il-1β*, P<0.0001; *Nos2*, P<0.01; *Mmp9*, P<0.0001. **Figures 3E–G**). Consistent with the qRT-PCR results, Western blot analysis revealed that R428 treatment inhibited Axl phosphorylation and attenuated IL-1β, NOS2, and MMP9 expression. rmGas6 promoted Axl phosphorylation, and increased IL-1β, NOS2, and MMP9 in M1-like macrophages (**Figures 3H–J**).

LPS/IFN-γ-primed THP-1 cells were treated with vehicle, R428 and rGas6. R428 treatment showed a lower level of pAxl (P<0.01), while the levels of Gas6 and Axl did not show a significant difference, and the secretion of IL-1β was attenuated (P<0.01). In contrast, Gas6 treatment in LPS/IFN-γ-primed THP-1 cells enhanced pAxl (P<0.01) and the secretion of IL-1β (P<0.05) without changing Axl. Gas6 plus R428 treatment inhibited pAxl (P<0.01) and IL-1β (P<0.01) secretion, as shown in **Supplementary Figure S2**.

### STAT1 knockdown abolished the promoting effect of Axl on M1 polarization

**Figure 4:**
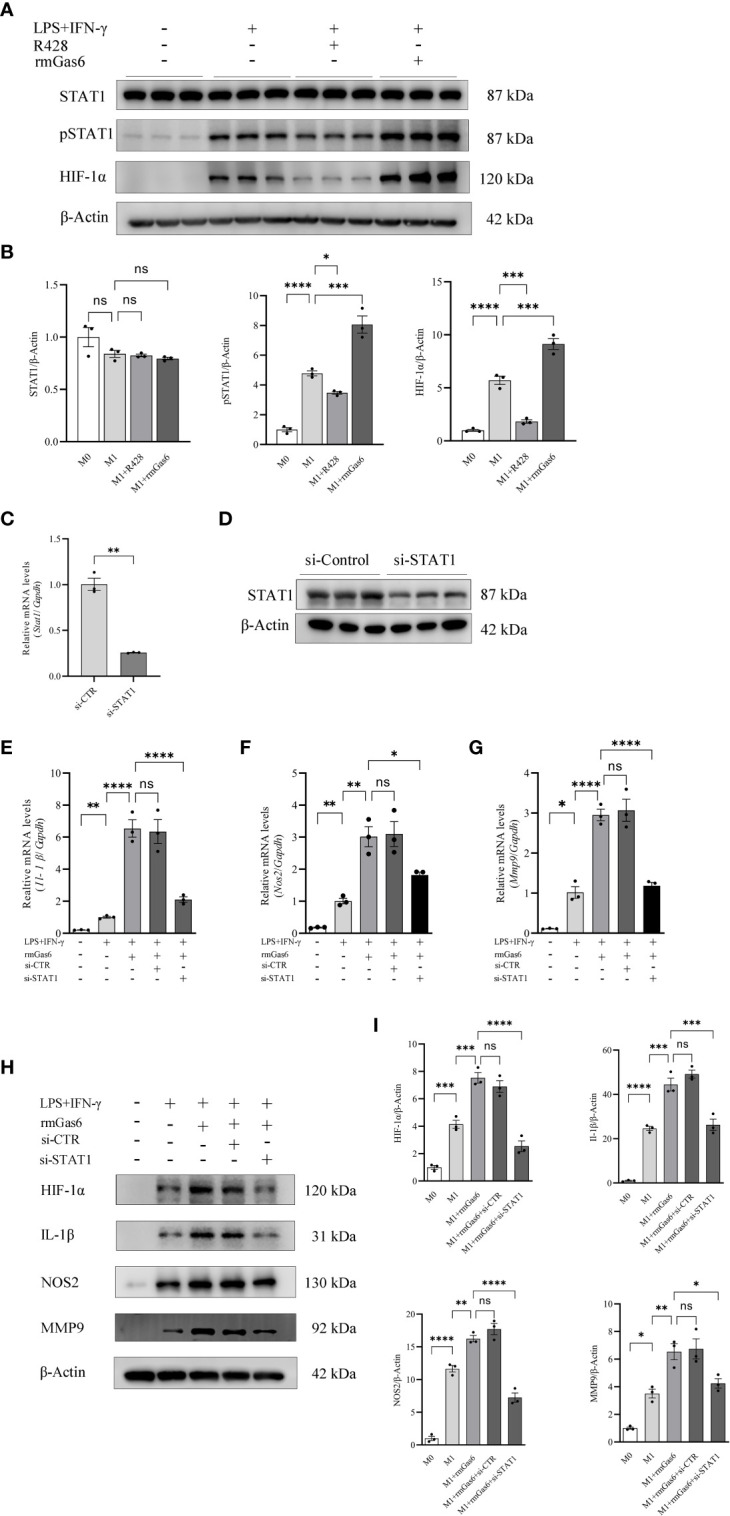
Axl skewed macrophage polarization toward M1 *via* STAT1/HIF-1α activation. A. STAT1 phosphorylation and HIF-1α activation measured by western blot 6 h after incubation with R428 or rmGas6 **(A, B)**. Verification of the efficiency of STAT1 knockdown at the mRNA **(C)** and protein levels **(D)**. The effects of STAT1 knockdown on *IL-1β*, *Nos2*, and *MMP9* secretion were measured by qRT-PCR **(E—G)** and western blotting in rmGas6-treated BMDMs **(H, I)**. ns, not significant, *P<0.05, ^**^P< 0.01, ^***^P< 0.001, ^****^P< 0.0001.

We found that R428 treatment inhibited the phosphorylation of STAT1 and expression of HIF-1α, while the phosphorylation of STAT1 and level of HIF-1α were promoted by rmGas6 in M1-like macrophages (**Figures 4A, B**). To illuminate the role of STAT1 in Axl-mediated macrophage polarization, the STAT1 level was knocked down *via* STAT1-specific siRNA, and the efficiency of knockdown was confirmed at the mRNA (P<0.01) and protein levels (**Figures 4C, D**). Compared with the si-CTR group, STAT1 knockdown attenuated *Il-1β* (P<0.001), *Nos2* (P<0.05), and *Mmp9* (P<0.0001) in rmGas6-treated M1 macrophages (**Figures 4E–G**). Western blot analysis showed the same result, as well as HIF-1α decline (**Figures 4H, I**). These results revealed that the effect of Axl on M1-like macrophage polarization was abolished by STAT1 knockdown.

### Activated Axl promotes intracranial aneurysm rupture in mice

**Figure 5:**
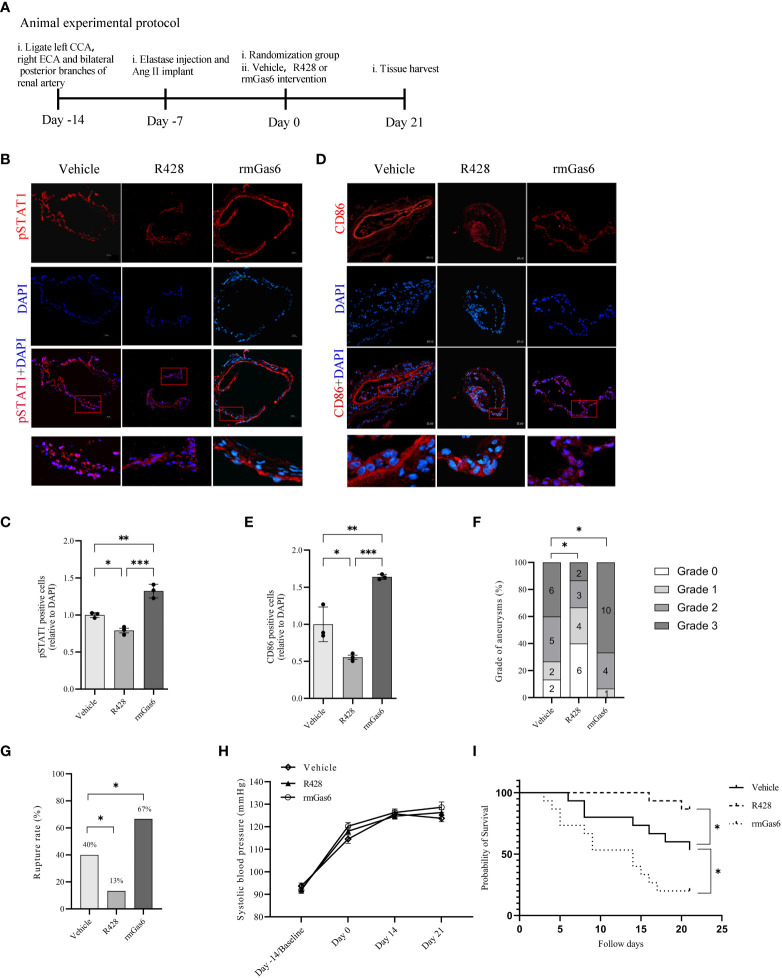
Axl promoted intracranial aneurysm progression and rupture in mouse models. **(A)**, Animal treatment protocol. **(B, C)**, Representative immunofluorescent images and quantitative analysis of pSTAT1 (red)-positive cells in unruptured aneurysms treated as indicated. **(D, E)**, Representative immunofluorescent images and quantitative analysis of CD86 (red)-positive cells in unruptured aneurysms treated as indicated. Scale bar=20 μm. **(F)**, Grade of intracranial aneurysm progression. **(G)**, Incidence of intracranial aneurysm rupture. **(H)**, Systolic blood pressure between groups. **(I)**, Symptom-free curve (log-rank analysis curve) (n=15). ^*^P < 0.05, ^**^P < 0.01, ***P< 0.001.

IA mice were designed and treated with vehicle, R428, and rmGas6 in a flowchart (**Figure 5A**). Compared with vehicle treatment, R428 treatment inhibited the phosphorylation of STAT1 (39.0% vs. 30.8%, P<0.01. **Figures 5B, C**) and significantly decreased M1-like macrophage infiltration in aneurysm walls (44.7% vs. 24.7%, P<0.05. **Figures 5D, E**). However, rmGas6 treatment boosted the phosphorylation of STAT1 (51.6% vs. 39.0%, P<0.01. **Figures 5B, C**) and M1-like macrophage infiltration (44.7% vs. 73.0%, P<0.01. **Figures 5D, E**).

The analysis revealed different compositions of pathological grades among the three groups (R428 vs. vehicle, n=15, P<0.05; rmGas6 vs. vehicle, n=15, P<0.05. **Figure 5F**). Meanwhile, R428 treatment significantly decreased the rate of IA rupture compared with that of the vehicle group (13% vs. 40%, n=15, P<0.05), which was reversed by rmGas6 treatment (67% vs. 40%, n=15; P<0.05. **Figure 5G**). Systolic blood pressure among the groups did not show differences and increased slowly since the induction of the model (group factor, P=0.2743; time factor, P<0.0001; no interaction effect. **Figure 5H**). Similarly, the symptom-free survival of mice was significantly increased in the R428 treatment group (P<0.05, n=15) and decreased in the rmGas6 treatment group (P<0.05, n=15. **Figure 5I**).

## Discussion

In this study, we found that Axl exacerbated the process of IA rupture by increasing M1 macrophage infiltration in aneurysm tissue and promoting proinflammatory responses in mice. Treatment with R428 to inhibit Axl phosphorylation significantly decreased the rate of IA rupture in mice. rmGas6 treatment promoted Axl phosphorylation and increased the rate of IA rupture. In addition, inhibition of Axl phosphorylation decreased STAT1 phosphorylation, attenuating M1 macrophage polarization *in vivo*. Mechanistically, Axl activation enhanced the STAT1/HIF-1α signaling pathway to promote macrophage polarization toward M1, which could be abolished by STAT1 knockdown. These findings potentially provide a new therapeutic target and may facilitate pharmacological treatment for IA.

Our results showed more CD86^+^ M1 macrophages in ruptured IAs than in unruptured IAs. Additionally, R428, an Axl-specific inhibitor, significantly decreased the infiltration of M1 macrophages into the artery wall and the occurrence of IA rupture. Our findings are consistent with other studies that suggest that macrophage polarization is associated with IA rupture. Macrophage infiltration is critical for aneurysm formation and rupture, and macrophage-mediated inflammation is a key biological pathway for IA rupture (22). Hasan et al. found an imbalance of M1/M2 macrophages and a trend toward M1 macrophages in ruptured IAs compared with unruptured IAs (10). There was a lower incidence of IA in a murine model due to macrophage depletion by clodronate liposomes and macrophage dysfunction by MCP-1 knockout (7). Macrophages infiltrate into intracranial arterial walls across endothelial cells and secrete proinflammatory cytokines and metalloproteinases (MMPs), such as MMP-2 and MMP-9, to disassemble the collagen matrix, which can lead to the destruction of the arterial wall and rupture (23).

We found that Axl expression was upregulated in unruptured IAs and further upregulated in ruptured IAs compared with normal arteries. These results indicated that Axl is involved in IA rupture. Meanwhile, the double immunofluorescence anti-Axl and anti-F4/80 antibodies, a macrophage-specific marker, in IA sections and Axl colocalized with F4/80^+^ cells in mouse IA sections showed the relationship between Axl expression and macrophage infiltration. We also found that smooth muscle cells in mouse aneurysms expressed AXL in contrast to those in normal vessels. Okada et al. (24) have shown that BMDMs are the major source of M1 macrophage infiltration into IA regions. These findings suggest that Axl may modulate M1 macrophage infiltration into the aneurysm wall of mice.

We found that LPS/IFN-γ promoted Axl expression and phosphorylation in BMDMs and THP-1 macrophages. Axl depends on Gas6 for its activation, and Gas6 requires Axl for its stable maintenance *in vivo* (17). Endogenous Gas6 levels may influence AXL receptor activation. rmGas6 is the specific ligand for Axl activation. We also found that R428 treatment had no effect on Gas6 expression in LPS/IFN-γ-primed THP-1 cells. R428 only or R428 plus recombinant Gas6 inhibited Axl activation. These results suggested that R428 inhibited Axl phosphorylation independently of added exogenous rGas6.

Upon binding with the ligand Gas6, Axl dimerizes and becomes phosphorylated, leading to the activation of downstream mitogen-activated protein kinase (MAPK) (25–27). Both LPS and IL-4 promote Axl expression, and the activation of Axl receptors occurs in BMDMs after stimulation with LPS to activate TLR receptors (17, 28). It would be interesting to investigate whether AXL activation occurs by cross-activation with other AXL receptor units or another heterodimeric receptor in further research. BMDMs were stimulated with LPS/IFN-γ to polarize toward M1 macrophages to mimic the inflammatory responses of IA. Axl expression was significantly increased by LPS/IFN-γ and colabeled with F4/80. Axl boosting in the intracranial aneurysmal inflammation-related microenvironment might be associated with M1 macrophage polarization.

We also found an explicit effect of Axl on macrophage polarization after LPS/IFN-γ stimulation. Both qRT-PCR and Western blotting showed that R428 inhibited Axl phosphorylation and attenuated IL-1β, NOS2, and MMP-9 in M1 macrophages. Conversely, the rmGas6 treatment had the opposite effect. These findings suggest that inhibition of Axl phosphorylation significantly attenuates M1 macrophage polarization and the production of IL-1β, NOS2 and MMP9. Our results are consistent with those of previous studies. The activation of Axl augments the expression and secretion of proinflammatory cytokines such as IL-1β, IL6, and TNFα in both macrophages and dendritic cells *via* the NLRP3 inflammasome and caspase-1, which can be enhanced by Gas6 and abolished by R428 (25, 28). Additionally, Axl augmented IL-1β and IL-6 production in LPS-primed Kupffer cells (26). IL-1β is important for the progression of intracranial aneurysms and acts as a representative cytokine secreted by M1 macrophages (27, 29). Macrophage-derived MMP-9 promotes intracranial aneurysm rupture (23). NOS2 is used to define classically activated M1 macrophages.

We found that R428 inhibited the levels of pSTAT1 and HIF-1α in M1 macrophages, while rmGas6 boosted them. STAT1 knockdown caused the downregulation of HIF-1α and inflammatory factors, including IL-1β, NOS2 and MMP9. STAT1 is one of the key regulators of inflammation and is important for macrophage polarization (30). HIF-1α positively regulates proinflammatory cytokines in response to LPS (31). These findings suggest that STAT1/HIF-1α might mediate the promotion of M1 macrophage polarization by Axl.

Axl signaling in macrophages exacerbated inflammation of the intracranial artery wall through STAT1/HIF-1α signaling. The effect of Axl was alleviated by STAT1 knockdown, emphasizing the key role of STAT1 in Axl signaling. Axl signaling promotes the phosphorylation and activation of STAT1, which is an important signaling molecule for the inflammatory response (12, 32). In addition, HIF-1α has been reported as the transcription factor of IL1β and is stimulated by STAT1 (33, 34). Furthermore, DeBerge et al. (28) found that the effect of Axl was HIF-1α dependent rather than NF-κB dependent. Both STAT1 and HIF-1α were linked to M1 macrophage activation within the M1/M2 macrophage polarization paradigm and served downstream of Axl signaling.

This study has some limitations. First, the animal model may not be sufficient to accurately depict the biological process of spontaneous IA rupture. Aneurysms were induced and did not spontaneously form in our models. Although the phenotype and presentation of aneurysm rupture mimic those of human intracranial aneurysms (35), it is difficult to definitively confirm the time course of Axl expression and M1 macrophage polarization during spontaneous IA rupture. Second, this study focused on STAT1/HIF-1α signaling but did not explore other signaling pathways. Axl-mediated efferocytosis and autophagy play a protective role in stimulating autophagy to reduce the release of inflammasomes in the innate immune system during self-limiting inflammation (36–38). Third, human intracranial aneurysm tissues are difficult to obtain because most aneurysms are treated with endovascular coiling. Although we found that R428 treatment inhibits AXL activation in LPS/IFN-γ-primed THP-1 cells, we did not verify this in human primary M1 macrophages *in vitro* which may provide additional translational information for our work. The comprehensive effect of Axl might be complex in intracranial aneurysms. Nevertheless, we clarified the mechanism by which Axl exacerbates macrophage polarization to the M1 phenotype *via* STAT1/HIF-1α signaling.

## Conclusions

The activation of Axl played a detrimental role in the destruction of the arterial wall and the rupture of the IA and promoted M1 macrophage polarization and infiltration into the arterial wall by enhancing STAT1/HIF-1α signaling. These findings suggest that the inhibition of Axl might be a potential strategy for the prevention of IA rupture. Axl plays an essential role in IA rupture and might be a potential therapeutic target for IAs.

## Data availability statement

The original contributions presented in the study are included in the article/**Supplementary Material**. Further inquiries can be directed to the corresponding authors.

## Ethics statement

The animal study was reviewed and approved by the Institutional Animal Care and Use Committee of Shanghai Jiaotong University.

## Author contributions

Conceptualization, HY and BZ; statistical analysis, ZZ and XZ; Methodology, YH and GL. Writing original draft, YH and GL; Writing, review & editing, BZ; All authors have reviewed and approved the version of the manuscript.
