# Supplementary material for: Axl promotes intracranial aneurysm rupture by regulating macrophage polarization toward M1 via STAT1/HIF-1α
Source: Front Immunol. 2023 May 8;14:1158758. doi: 10.3389/fimmu.2023.1158758 (PMC10200875; doi:10.3389/fimmu.2023.1158758)
Supplement: Supplementary file 1 [file DataSheet_1.docx]

Supplementary materials

Fig. S1


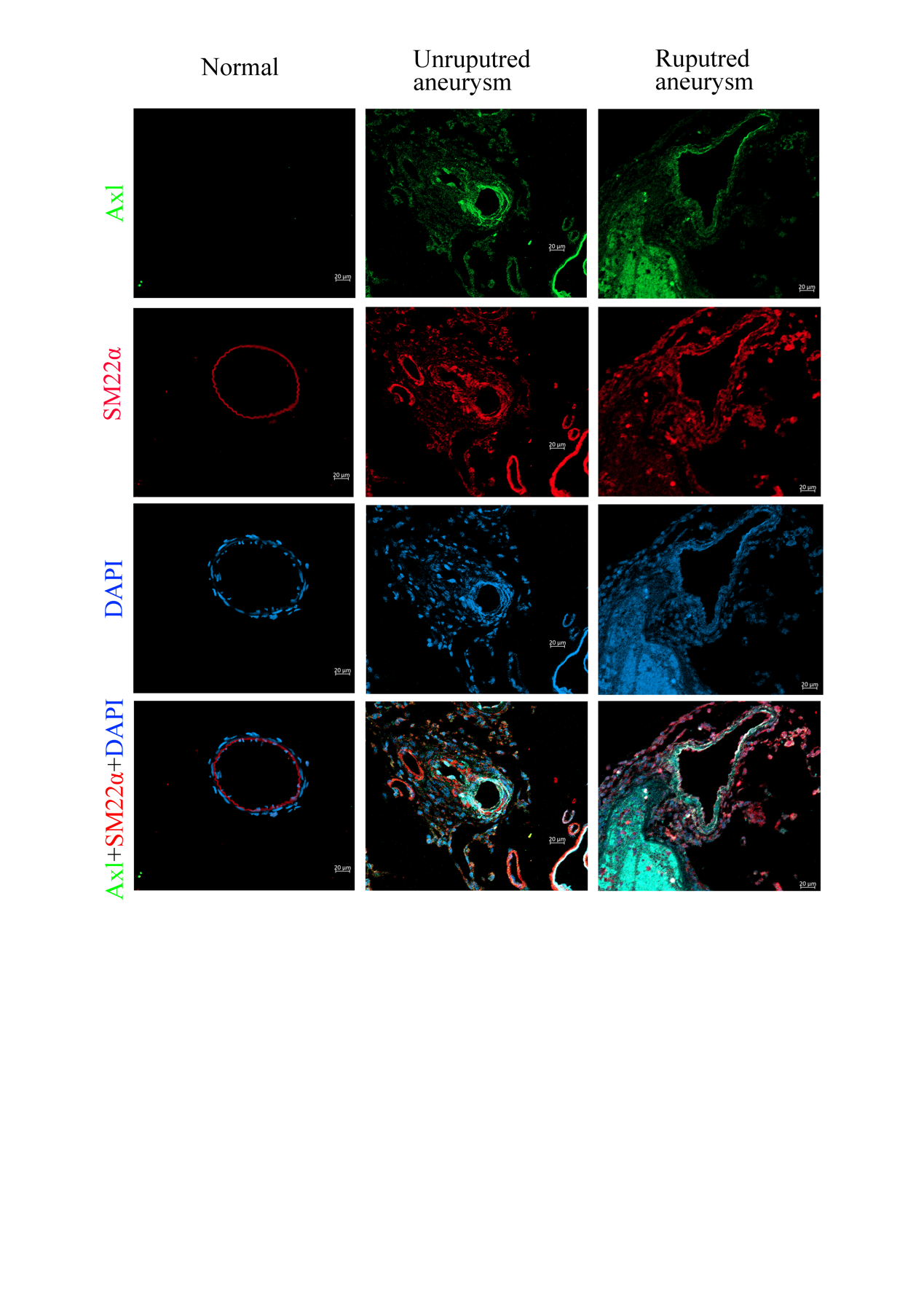


**Fig S1.Axl co-expressed with SM22****α in aneurysms.**

In normal artery, Axl (Green) positive cell was few but SM22α (Red) is obvious. In unruptured and ruptured aneurysms, Axl (Green) is increased and co-expressed with SM22α (Red).

**Supplementary materials**

**Fig. S2**


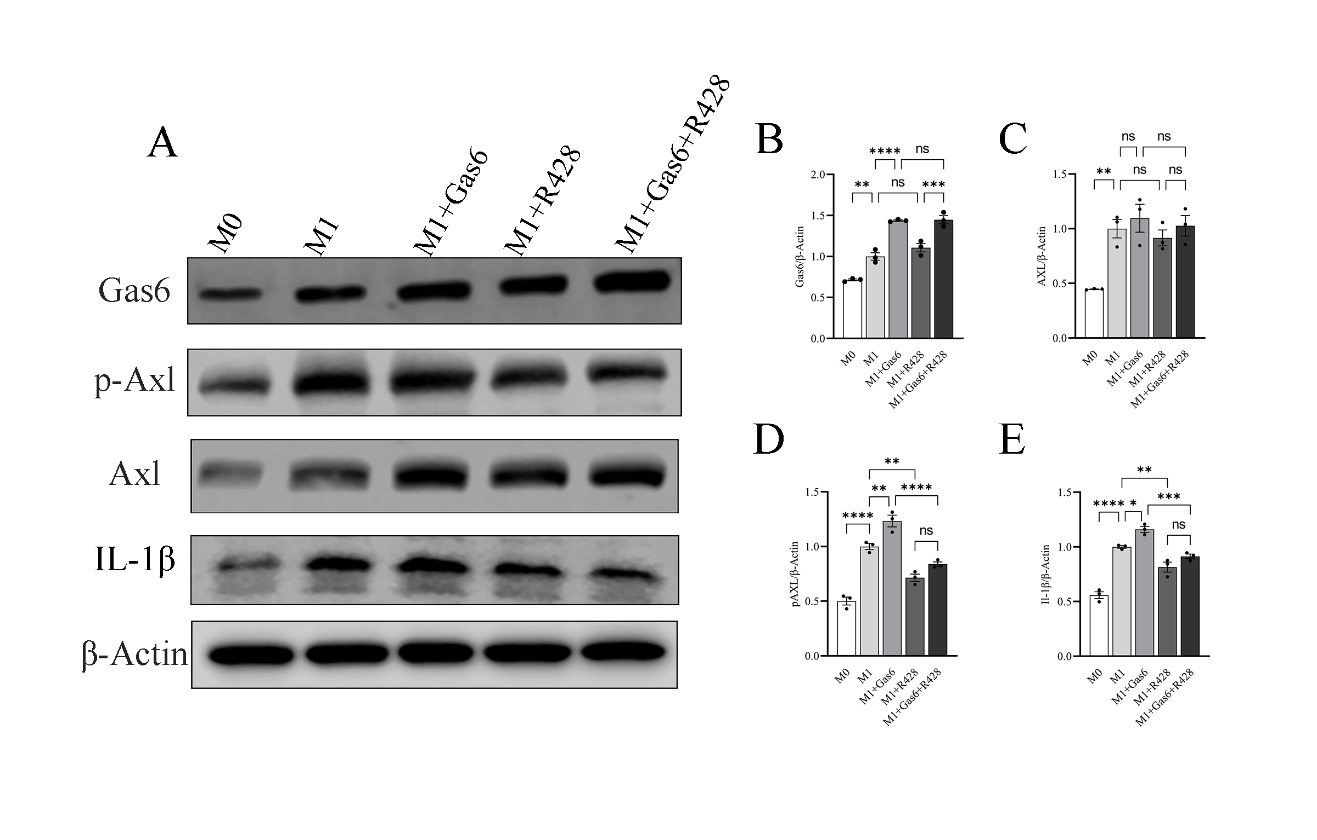


**Fig S2. R428 inhibited Axl phosphorylation and IL-1β expression independent of exogenous rGas6 in THP-1 cells.**

Expression of Gas6, Axl, and IL-1β and phosphorylation of Axl measured by western blot 6 h after incubation with Gas6, R428, or Gas6 plus R428 (A). Quantitative analysis of Gas6 (B), Axl (C), p-Axl (D), and IL-1β (E).
